# Efficacy and safety of traditional Chinese medicine injection with mecobalamin in treating diabetic peripheral neuropathy: A protocol for systematic review and meta-analysis

**DOI:** 10.1097/MD.0000000000023702

**Published:** 2021-01-08

**Authors:** Weihua Mai, Aisheng Wei, Xiaoxuan Lin, Funeng Wang, Jianhong Ye, Ping Chen

**Affiliations:** a Department of Endocrinology, Foshan Hospital of Traditional Chinese Medicine; b Department of Medicine, Zhangcha Hospital, Foshan, Guangdong Province, China.

**Keywords:** diabetic peripheral neuropathy, mecobalamin, meta-analysis, protocol, systematic review, traditional Chinese medicine injection

## Abstract

**Background:**

Diabetic peripheral neuropathy is a common complication of diabetes and the main cause of disability. At present, there is no specific therapeutic regimen. Mecobalamin is often used as a neurotrophic drug, and its long-term effects are not satisfactory when used alone. Clinical practice indicates that traditional Chinese medicine injection with mecobalamin has a therapeutic advantage in treating diabetic peripheral neuropathy while it lacks evidence-based medicine. In this scheme, the efficacy and safety of traditional Chinese medicine injection with mecobalamin in treating diabetic peripheral neuropathy has been studied.

**Methods:**

Computers were used to search the English database (PubMed, the Cochrane Library, Embase, Web of Science), and Chinese database (CNKI, Wanfang, CBMDISC, VIP). Besides, manual searching was conducted to search for Baidu Scholar, CHICTR, Google Scholar. During the establishment of the database to November 2020, a randomized controlled trial on traditional Chinese medicine injection with mecobalamin in treating diabetic peripheral neuropathy was conducted. There were 2 researchers independently conducting data extraction and quality evaluation of literature on the included studies, RevMan5.3 was performed for meta-analysis on the included literature.

**Results:**

In this study, the efficacy and safety of traditional Chinese medicine injection with mecobalamin in treating diabetic peripheral neuropathy was evaluated by the total effective rate, motor nerve conduction velocity, sensory nerve conduction velocity, adverse reactions, and glucose metabolism level.

**Conclusion:**

This study can provide an evidence-based basis on the clinical applications of traditional Chinese medicine injection with mecobalamin in the treatment of diabetic peripheral neuropathy.

**Ethics and dissemination:**

The study does not involve patient privacy or rights and does not require approval from an ethics committee. The results may be published in peer-reviewed journals or disseminated at relevant conferences.

**OSF Registration number:**

DOI 10.17605/OSF.IO/KPW5E.

## 1 Introduction

Diabetes is a chronic metabolic disease. In recent years, with the acceleration of population aging and the change of lifestyle, the incidence of it keeps an upward tendency.^[1]^ All kinds of serious complications can be led because hyperglycemia and metabolic disorders will damage the systemic tissues and organs.^[2]^ Diabetic peripheral neuropathy (DPN) which can cause many symptoms, such as pain, is the most common complication.^[3]^ An acute attack is defined as an alarm signal that protects the body, whereas it turns into a disease when it is chronic.^[4]^ As a chronic non-infectious disease, diabetic peripheral neuropathy is also known as symmetrical and peripheral polyneuropathy, leading to injury of vascular endothelial cell and hemodynamic changes.^[5]^ Relevant statistics show that the incidence rate of DPN in patients with diabetes can reach up to 65% to 90%, because the early onset symptoms are not obvious, the majority of patients have sensory disturbance in the lower extremities. They usually have some feelings such as cold body, numbness, or tingling in the extremities.^[6]^ After the disease's onset, the patient presents symmetrical pain accompanied by paresthesia. This kind of symptom progresses from the feet and hands to the proximal end. The patient presents joint lesions or ulceration in the lower extremity when the disease becomes severe, threatening the patient's limbs.^[7]^ Clinically, mecobalamin is a commonly used drug in treating peripheral neuropathy. It can participate in the methyl transformation of substances, promote lecithin synthesis in the medullary sinus, and the metabolism of protein, nucleic acid, and lipids, repair the impaired nerve tissue, and relieve the neurological sign of patients_._^[8]^ However, monotherapy with mecobalamin is not effective because the pathogenesis of DPN is complex. Currently, drug combination therapy is paid attention to in clinical practice.^[9]^

With the long history in preventing and treating chronic complications such as diabetes, traditional Chinese medicine has certain advantages in treating DPN. Traditional Chinese medicine categorizes DPN as severe pain and diabetes, the pathogenesis of DPN is mainly Yin deficiency and dryness-heat. The cause is long-time consumptive thirst, which leads to defect of qi and Yin, obstruction of blood flow, blood stasis, and stagnation leading to pain.^[10]^ Therefore, the treatment should be on the principle of activating pulse-beat and clearing heat, tonifying qi, and activating blood.^[11]^ Studies have reported that traditional Chinese medicine plays a special role in treating DPN.^[12]^ At present, traditional Chinese medicine injections for DPN have Danhong injection, Xuetong injection, Ginkgo leaf injection, Danshen ligustrazin for injection, and so on.^[13]^ Studies have shown a significant effect on treating DPN by combining traditional Chinese and western medicine can effectively relieve patients’ clinical symptoms and significantly improve nerve conduction velocity.^[14]^ However, a certain difference among the different studies was different, and the sample size of these studies is small. This study conducted a systematic review and meta-analysis of published randomized controlled trials to provide an evidence-based basis for treating diabetic peripheral neuropathy by traditional Chinese medicine injection with mecobalamin.

## 2 Methods

### 2.1 Protocol register

This systematic review protocol and meta-analysis have been drafted under the guidance of the preferred reporting items for systematic reviews and meta-analysis protocols (PRISMA-P). And, the frame of this study has already registered in the open science framework (OSF) (registration number: DOI 10.17605/OSF.IO/KPW5E).

### 2.2 Ethics

The study does not involve patient privacy or rights and does not require approval from an ethics committee. The results may be published in peer-reviewed journals or disseminated at relevant conferences.

### 2.3 Eligibility criteria

#### 2.3.1 Types of studies

We will collect all randomized controlled trials (RCTs) of Chinese herbal injections combined with mecobalamin in the treatment of diabetic neuropathy, regardless of their blinding, publication status or location, but only in Chinese and English.

#### 2.3.2 Object of study

Patients with diabetic peripheral neuropathy. There is no limitation on nationality, race, course of the disease, region, sex, age, disease time, and constitution.

#### 2.3.3 Intervening measures

Traditional Chinese medicine injection with mecobalamin was used in the treatment group, and there was no limitation on the type, dosage, and course of treatment. Mecobalamin or traditional Chinese medicine injection was used in the control group. There was no limitation on the dosage, course of mecobalamin, and the type, dosage, course of traditional Chinese medicine injection.

#### 2.3.4 Outcome indicators

Total effective rate; motor nerve conduction velocity; sensory nerve conduction velocity; adverse reaction; glucose metabolism level; FPG (fasting plasma glucose); HbA1c (glycosylated hemoglobin).

### 2.4 Exclusion criteria

(1) Unable to acquire the full articles;
(2) Data lacks and cannot be obtained by contacting the author;
(3) The outcome indicators are inconsistent with that in this study;
(4) Patients with peripheral neuropathy caused by other reasons;
(5) Use other traditional Chinese medicine treatment methods in the treatment group, such as Tai Chi, acupuncture, and massage, etc.

### 2.5 Search strategy

As Chinese search terms, “Diabetic neuropathies,” “diabetic neuralgia,” “mecobalamin,” “Traditional Chinese medicine,” and “injection” were searched in Chinese databases such as Chinese National Knowledge Infrastructure (CNKI), China Biomedical Database (CBM), Wanfang Data, and VIP. As English search terms, “Diabetic neuropathies,” “Diabetic neuralgia,” “Traditional Chinese medicine injection,” “Mecobalamin” were searched in English database such as PubMed, Web of Science, Embase, the Cochrane Library, and conducted manual retrieval on Google academic, Baidu Scholar, SCI-HUB. The retrieval time was from the establishment of the database to November 2020. Domestic and foreign literature about treating diabetic peripheral neuropathy by traditional Chinese medicine injection with mecobalamin was collected. Take PubMed as an example. The search strategy is shown in Table 1.

**Table 1:** Search strategy of PubMed database.

Number	Search terms
#1	Diabetic neuropathies [MeSH]
#2	Diabetic neuropathies [Title/Abstract]
#3	Diabetic peripheral neuropathy [Title/Abstract]
#4	Diabetic neuralgia [Title/Abstract]
#5	#1 OR #2 OR #3 OR #4
#6	Traditional Chinese medicine injection [Title/Abstract]
#7	Injection of Chinese medicine [Title/Abstract]
#8	#6 OR #7
#9	Mecobalamin [MeSH]
#10	Mecobalamin [Title/Abstract]
#11	#9 OR #10
#12	#5AND #8AND #11

### 2.6 Data filtering and extraction

**Figure 1:**
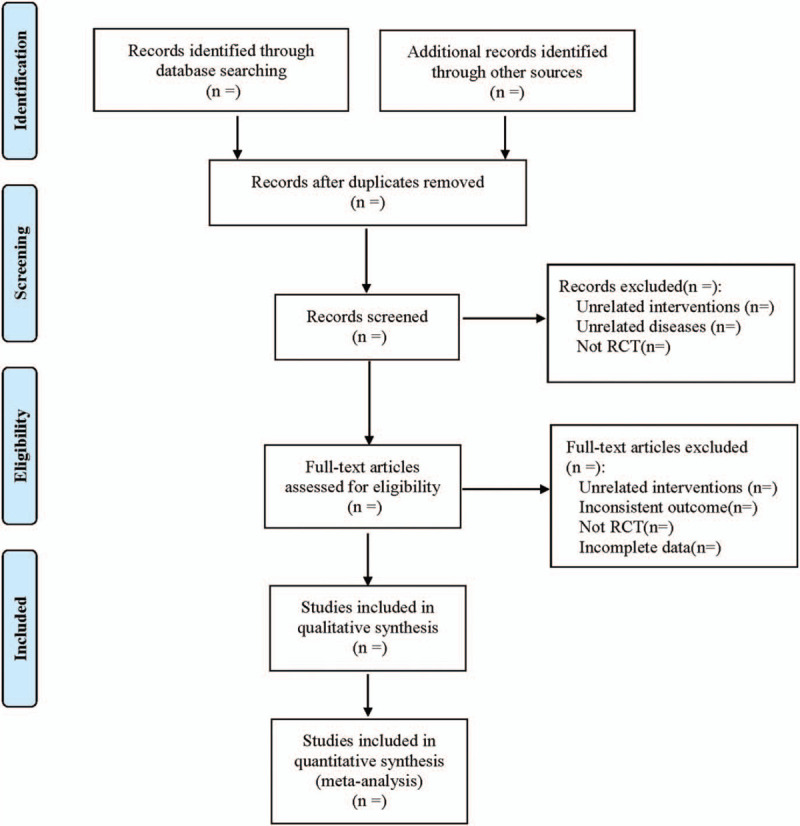
Flow diagram.

According to the methods of inclusion in the 5.0 version of Cochrane Manual for Systematic Evaluation of Interventions, 2 researchers imported the literature into EndNote X7 based on inclusion and exclusion criteria, then set up groups and duplicated checking. The literature was screened by reading the title and abstract and then screened it again after reading the full text. If there were a disagreement, a third researcher would participate in the discussion. Meanwhile, Excel 2013 was used to extract relevant information, including the first author, year of publication; basic information of the subjects: physique, age, height, weight, course of the disease, sex, sample size; intervention methods of the treatment group and the control group: mecobalamin with traditional Chinese medicine injection was adopted in the treatment group, mecobalamin or traditional Chinese medicine injection was adopted in the control group; outcome indicator: total effective rate, motor nerve conduction velocity, sensory nerve conduction velocity, adverse reactions, FPG (fasting blood glucose), HbA1c (glycosylated hemoglobin), etc.; quality evaluation of literature; adverse events, follow-up condition; methodological information of literature. The literature screening process is shown in Fig. 1.

### 2.7 Quality evaluation of literature

The Cochrane collaboration's tool for assessing the risk of bias in the Review Manager5.3 software assessed risk bias of included studies. According to the random sequence generation's performance, allocation concealment, blinding of participants and personnel, blinding of outcome assessment, incomplete outcome data, selective reporting, and other bias, 2 researchers gave low risk, unclear risk, high risk term by term, and checked after completion. If there were any differences, the discussion would be made. If there were no agreement, the third party would participate in this discussion.

### 2.8 Statistical analysis

Meta-analysis was performed by RevMan5.3 software. Relative ratio (RR) was used to present the binary variable. For continuous outcomes, if the measuring tool were consistent with the measurement unit, the weighted mean difference (WMD) would be used to present it; if the measuring tool were not consistent with the measurement unit, the standard mean difference (SMD) would be used to present it. All of these were present by a 95% confidence interval (CI). Chi-squared and *I*^2^ value judged heterogeneity. If *P* *≥* .1, *I*^2^ ≤ 50% indicated the heterogeneity was low, a meta-analysis would be conducted by the fixed effect model. If *P* < .1, *I*^2^ > 50% indicated heterogeneity among studies, the source of heterogeneity would be analyzed. Clinical heterogeneity was performed by subgroup analysis. If there were no obvious clinical heterogeneity or methodological heterogeneity, statistical heterogeneity would be considered, and a random-effects model would be conducted. If the clinical heterogeneity was too obvious, and subgroup analysis could not be conducted, the descriptive analysis would be performed but not a meta-analysis.

#### 2.8.1 Dealing with missing data

If it lacked data, please contact the corresponding author or the first author to acquire the exact data. If it were impossible to contact authors and acquire relevant data, the descriptive analysis would be conducted instead of meta-analysis.

#### 2.8.2 Subgroup analysis

The subgroup analysis was performed according to the sex or age, the type of traditional Chinese medicine injection, and the course of Chinese medicine injection.

#### 2.8.3 Sensitivity analysis

A sensitivity analysis was conducted to analyze all of the outcome indicators to judge outcome indicators’ stability.

#### 2.8.4 Assessment of reporting biases

Funnel plots were performed to evaluate publication bias if no fewer than 10 studies were included in an outcome measure. Besides, Egger test and Begg test were used for the evaluation of potential publication bias.

#### 2.8.5 Quality evaluation of evidence

Based on the international GRADE system, evidence quality and recommendation levels were evaluated. According to the reliability of the assessment results of the curative effect of traditional Chinese medicine injection with mecobalamin in treating diabetic neuropathy, the hierarchy of evidence quality was divided into high quality, medium quality, low quality, and deficient quality. The recommendation level could be divided into strong and weak recommendation levels, which respectively indicated that the advantages of intervening measure outweigh its disadvantages and the advantages of intervening measure likely outweigh its disadvantages.

## 3 Discussion

As one of the most common complications of diabetes, DPN is a progressive degenerative disease in the peripheral nervous system which needs high energy.^[15]^ DPN has many clinical manifestations, including sensory disorder and dyskinesia, leading to morbidity, disability, and mortality, such as lower limb infections, muscle atrophy, ulcers, and amputations. All of these will seriously endanger patients’ physical and psychological health and life safety.^[16,17]^ There is no specific treatment clinically since the etiology and pathogenesis are unclear. At present, based on normal blood glucose, drugs can reverse neuropathy are often used for treatment, such as alprostadil, mecobalamin, and so on. As an endogenous vitamin B12 commonly used clinically, Mecobalamine can participate in the body's carbon cycle and transmethylase reaction. Folic acid can promote the synthesis of proteins and lecithin in nerve cells and the regeneration of neuronal axons and axoplasmic transport, then regulate the metabolism of nucleic acid. Besides, mecobalamin affects synapses, increases acetylcholine level between synapses, promotes nerve impulse transmission, and speeds up the conduction between neurons. Compared with other nerve drugs, it is easier to penetrate neurons.^[18]^ However, studies have shown that it is more effective to combine Chinese medicine with western medicine for DPN than Western medicine only.^[19]^ According to traditional Chinese medicine theory, diabetic peripheral neuropathy belongs to the category of quenching thirst. As the main branch of traditional Chinese medicine, Chinese herbal medicine is widely used in treating diseases. The flexible adjustment of herbs makes each prescription perform its various functions. Combined with the holistic theory of traditional Chinese medicine, herbs reduce drug–drug and drug–condition interactions. In recent years, traditional Chinese medicine has played an increasingly important role in treating complex pathogenesis and symptoms such as DPN.^[20]^ The mechanism of traditional Chinese medicine in treating DPN is protecting pancreatic cells, improving blood glucose and lipids, and delaying the disease's progression by antioxidant therapy.^[21,22]^ Studies show that using three traditional Chinese medicine injections, including Danhong injection, Ginkgo leaf injection, and Danshen Chuanxiongqin injection, are better than using mecobalamin only in the adjuvant treatment of DPN.^[23]^ The main components are tanshinone, Salviol, ginkgo biloba extract of Safflower, etc. Modern studies show that tanshinone, B Salvianolic acid can cure atherosclerosis, reduce blood viscosity, improve microcirculation, and eliminate oxygen radicals. Carthamin yellow in safflower has various effects, such as significantly inhibiting platelet aggregation, reducing the length and weight of thrombus, reducing blood fat, and delaying arterial plaque formation.^[24]^ The ginkgo biloba extract in ginkgo leaf injection affects scavenging oxygen free radicals, regulating various antioxidant enzymes, increasing the local blood volume, and inhibiting platelet aggregation by expanding capillaries so that improving microcirculation and treating DPN.^[25]^

In conclusion, compared with using medicine only and conducting basic treatment, it is more accurate to combine traditional Chinese medicine injection with mecobalamin to treat DPN. This scheme can significantly reduce blood glucose and blood lipid levels and improve hypercoagulable state and microcirculation. It can also promote nerve repair and improve the excitability and nerve conduction velocity of sensory and motor nerves.

However, due to the sample size being small and no observation of the long-term treatment's safety, large-sample, multicenter, mutually supportive, and multi-regional RCTs should be adopted based on the requirements of evidence-based medicine in the future. And long-term follow-up should be performed to verify the advantages of traditional Chinese medicine injection with mecobalamin in treating DPN.

## Author contributions

**Data curation:** Weihua Mai, Aisheng Wei.

**Funding acquisition:** Weihua Mai.

**Software:** Funeng Wang, Jianhong Ye.

**Supervision:** Xiaoxuan Lin.

**Writing – original draft:** Weihua Mai, Aisheng Wei.

**Writing – review & editing:** Weihua Mai, Ping Chen.
